# Role of the Aryl Hydrocarbon Receptor in Environmentally Induced Skin Aging and Skin Carcinogenesis

**DOI:** 10.3390/ijms20236005

**Published:** 2019-11-28

**Authors:** Christian Vogeley, Charlotte Esser, Thomas Tüting, Jean Krutmann, Thomas Haarmann-Stemmann

**Affiliations:** 1 IUF–Leibniz-Research Institute for Environmental Medicine, 40225 Düsseldorf, Germany; christian.vogeley@iuf-duesseldorf.de (C.V.); Charlotte.esser@iuf-duesseldorf.de (C.E.); jean.krutmann@iuf-duesseldorf.de (J.K.); 2 Laboratory of Experimental Dermatology, Department of Dermatology, Otto-von-Guericke University, 39120 Magdeburg, Germany; thomas.tueting@med.ovgu.de; 3 Medical Faculty, Heinrich-Heine University, 40225 Düsseldorf, Germany

**Keywords:** DNA damage, extracellular matrix, extrinsic skin aging, melanoma, particulate matter, pigmentation, polycyclic aromatic hydrocarbons, squamous cell carcinoma, UV radiation

## Abstract

The skin is constantly exposed to a variety of environmental threats, including solar electromagnetic radiation, microbes, airborne particulate matter, and chemicals. Acute exposure to these environmental factors results in the activation of different signaling pathways that orchestrate adaptive stress responses to maintain cell and tissue homeostasis. Chronic exposure of skin to these factors, however, may lead to the accumulation of damaged macromolecules and loss of cell and tissue integrity, which, over time, may facilitate aging processes and the development of aging-related malignancies. One transcription factor that is expressed in all cutaneous cells and activated by various environmental stressors, including dioxins, polycyclic aromatic hydrocarbons, and ultraviolet radiation, is the aryl hydrocarbon receptor (AHR). By regulating keratinocyte proliferation and differentiation, epidermal barrier function, melanogenesis, and immunity, a certain degree of AHR activity is critical to maintain skin integrity and to adapt to acute stress situations. In contrast, a chronic activation of cutaneous AHR signaling critically contributes to premature aging and the development of neoplasms by affecting metabolism, extracellular matrix remodeling, inflammation, pigmentation, DNA repair, and apoptosis. This article provides an overview of the detrimental effects associated with sustained AHR activity in chronically stressed skin and pinpoints AHR as a promising target for chemoprevention.

## 1. Introduction

Skin aging is driven by internal and external factors and respective maladaptive responses of the human body. The totality of these factors, to which human beings are exposed from conception to death was recently defined as the “skin aging exposome” [1]. Approximately one-half of this exposome consists of environmental factors and conditions, including solar radiation, air pollution, tobacco smoke, and temperature. These factors foster degenerative processes in the tissue leading to the development of the characteristic traits of extrinsic skin aging, i.e., coarse wrinkles and pigment spots (lentigines) [1,2,3]. In addition, life-long accumulation of macromolecular damages induced by these environmental stressors, in particular, ultraviolet (UV) radiation and polycyclic aromatic hydrocarbons (PAHs), holds another risk, that is the development of various skin malignancies, including malignant melanoma and keratinocyte-derived basal cell and squamous cell carcinomas [4,5,6]. The incidence of skin cancer is currently dramatically increasing, fueled by the continuously growing number of elderly individuals in the general population and the unbroken popularity of tanned skin among younger generations [7,8,9,10].

The aryl hydrocarbon receptor (AHR) is a cytosolic transcription factor that is activated by a broad variety of exogenous and endogenous small molecular weight compounds [11,12]. In the mid-1970s, AHR was identified as a 2,3,7,8-tetrachlorodibenzo-*p*-dioxin (TCDD)-binding receptor protein that regulates xenobiotic metabolism and critically shapes the toxicity of dioxins, polycyclic aromatic hydrocarbons (PAHs), and related persistent organic pollutants [12]. Meanwhile, a large body of literature provides evidence that AHR also binds various endogenously generated as well as plant- and microbiota-derived compounds, such as indoles, eicosanoids, polyphenols, and phenazines [11,13], and is involved in the regulation of various physiological and pathophysiological processes, including the development, maintenance, and function of certain immune cell populations [11,14].

**Figure 1:**
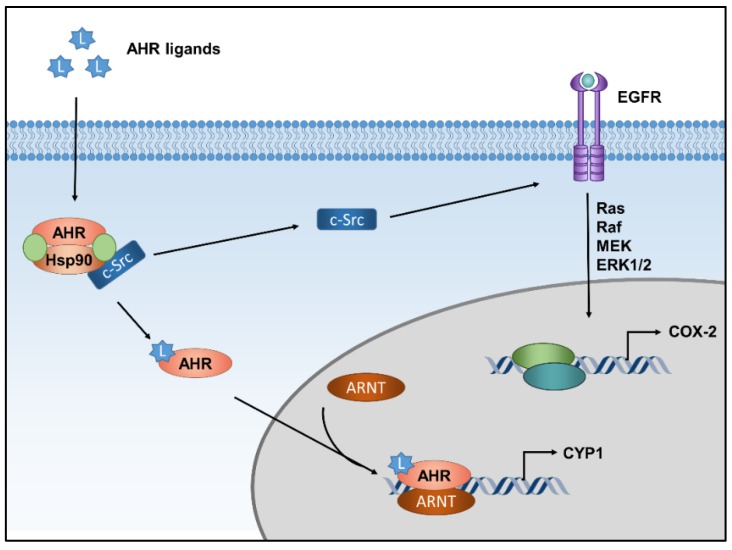
AHR-dependent signaling pathways. In its inactive state, AHR is part of a multiprotein complex consisting of different chaperone molecules and, possibly, tyrosine kinase c-Src. Upon ligand binding, the complex dissociates and AHR translocates into the nucleus, where it dimerizes with ARNT to form a transcriptionally active complex and induces the expression of target genes, for instance encoding CYP1 isoforms. In addition to this canonical signaling pathway, the ligand-driven dissociation of the cytosolic AHR multiportion complex stimulates c-Src activity, which is followed by activation of EGFR and downstream MAPK signaling, resulting in the transcriptional induction of another set of genes, such as cyclooxygenase-2 (COX-2).

In the absence of a ligand, AHR is part of a cytosolic multiprotein complex, consisting of two heat-shock protein 90 molecules, the AHR-interacting protein and the co-chaperone p23 [12]. In addition, an association of the complex with the soluble tyrosine kinase c-Src is discussed [15]. Binding of a ligand to the AHR protein causes conformational changes that lead to a dissociation of the protein complex and the nuclear translocation of AHR. In the nucleus, AHR dimerizes with its partner protein AHR nuclear translocator (ARNT) and binds to xenobiotic-responsive elements in the enhancer region of target genes to recruit RNA polymerase II and induces their transcription (Figure 1) [12]. The prototype target gene, which is often regarded as a biomarker for the induction of AHR activity, encodes for the drug-metabolizing monooxygenase cytochrome P450 (CYP) 1A1 [13]. In addition to this so-called canonical AHR pathway, the ligand-driven dissociation of the cytosolic multiprotein complex may result in the activation of the tyrosine kinase c-Src, which subsequently can activate epidermal growth factor receptor (EGFR) and downstream mitogen-activated protein kinase (MAPK) signal transduction (Figure 1) [15,16,17].

In addition, AHR may crosstalk with several other cellular pathways, including nuclear factor-kappa B (NF-κB) [18,19], hypoxia-inducible factor-1 [20,21] and estrogen receptor [22] signaling. AHR also interacts with the nuclear factor erythroid 2-related factor 2 antioxidant response pathway in manifold ways [23,24]. These non-canonical pathways and functions of AHR probably shape the responses towards AHR ligands contributing to the development of tissue-, cell- and microenvironment-specific effects.

AHR is expressed in all cutaneous subpopulations investigated so far [25] and contributes to a variety of physiological functions, including keratinocyte differentiation, epidermal barrier function, and melanogenesis [26,27,28]. In the epidermal compartment, ultraviolet (UV) radiation, in particular its UVB part (280−320 nm), is absorbed by the aromatic amino acid tryptophan, leading to the formation of photoproducts, such as 6-formylindolo(3,2-*b*)carbazole (FICZ) and 1-(1H-indol-3-yl)-9H-pyrido(3,4-*b*)indole, that bind with high affinity to AHR and induce downstream signaling responses [16,29,30]. Importantly, FICZ is detectable in human skin in vivo [31], and its metabolites can be found in urine samples from UV-exposed individuals [32]. Accordingly, in a placebo-controlled trial, we observed an AHR-dependent transcriptional induction of CYP1A1 and COX-2 in the skin of UVB-irradiated human individuals [33]. In addition, due to their lipophilic nature enabling a passive diffusion across cell membranes, PAHs present in tobacco smoke, ambient air pollution, and diet may not only penetrate the exposed barrier organs and activate AHR signaling therein, but also reach the circulation and thus secondary organs [34,35,36]. Hence, besides topical exposure [37], a systemic uptake of PAHs, dioxin-like chemicals or other AHR ligands can potentially affect AHR activity in the skin [37,38]. Finally, various AHR-binding metabolites produced by skin-residing bacteria [39,40,41] and yeasts [42,43] may shape cutaneous AHR signaling.

Beside its critical role in the development of chloracne, the hallmark of acute intoxication with dioxin-like chemicals in humans [44], AHR is involved in the pathogenesis of various other diseases and disorders of the skin, including atopic dermatitis, psoriasis, vitiligo, and cancer [45]. Interestingly, while exposure to several AHR agonists may foster the development and progression of certain skin diseases, such as atopic dermatitis [46,47,48], similar agents are being used for decades to treat these diseases [49,50]. This obvious discrepancy may be due to alterations in the microenvironment, e.g., healthy vs. inflamed skin, and respective differences in skin-residing immune cell populations or epidermal barrier function at the time of exposure [45] or to different chemical co-exposures and respective mixture effects [51]. Accordingly, depending on intention (prevention or therapy), duration and height of exposure to environmental, occupational and dietary factors, genetic predisposition, and type of skin disease, either activation or inhibition of cutaneous AHR activity may be beneficial [45].

In the following, we focus on cutaneous AHR signaling in chronic exposure scenarios and summarize how and to which extent respective signaling pathways contribute to maladaptive processes fostering extrinsic skin aging, i.e., the formation of coarse wrinkles and pigment spots, and the development of aging-associated malignancies.

## 2. AHR and Extrinsic Skin Aging

Chronic exposure of the skin to environmental factors, in particular, UV radiation, PAHs, and air pollution does not only pose the risk for the development of inflammatory and/or malignant diseases but also facilitates processes that drive premature aging of the barrier organ. The clinical hallmarks of extrinsic skin aging are the generation of coarse wrinkles and pigment irregularities [2,52]. It is commonly considered that ethnical differences in the expression of skin aging-related clinical features exist [53]. For example, a study comparing the progression of skin aging features in 160 Chinese and 160 French (age-matched) women, revealed the existence of ethnical alterations in skin aging. The generation of pigment spots was the dominating skin aging-related phenotype in Chinese women, whereas wrinkle formation dominated in the French study group. In the Chinese women, the onset of wrinkles seemed to be delayed by approximately 10 years and lacked linearity as compared to the French cohort [54]. Results from another epidemiological study indicate that the different manifestations of skin aging in Asian and Caucasian women might be, at least in part, due to differences in sun exposure and antioxidant plasma levels [55]. Importantly, all major environmental factors relevant for extrinsic skin aging, i.e., UV radiation [52], tobacco smoke [56,57], and air pollutants, especially PAH-rich particulate matter [58,59], are capable of activating AHR signaling in the skin. In fact, various molecular mechanisms and pathways by which an increased activity of cutaneous AHR may contribute to the generation of coarse wrinkles and lentigines (pigment spots) are discussed in the literature.

### 2.1. Extracellular Matrix Degradation and Wrinkle Formation

Coarse wrinkle formation results to a major extent from increased expression and activity of matrix metalloproteases (MMPs), which degrade the extracellular matrix (ECM), leading to loss of cutaneous elasticity and tensile strength. Dermal fibroblasts adhere to the ECM, in particular to collagen I fibrils, and synthesize ECM components to maintain its structural integrity [60]. During aging, fragmentation of collagen I fibrils results in reduced fibroblast spreading, attenuated synthesis of ECM molecules, and an enhanced production of MMPs [60,61]. Collagen I fibers are primarily degraded by MMP-1 and the resulting collagen I fragments serve as substrates for other MMPs, such as the gelatinases MMP-2 and MMP-9 [61]. As summarized by Ohtsuki and co-workers, several other MMPs may contribute to extrinsic skin aging as well [61]. Exposure to UV radiation [62] and tobacco smoke extract [63,64] is well-known to induce connective tissue damage by upregulating the expression of MMP-1 and MMP-3. In addition, these environmental stressors may also modulate transforming growth factor-β (TGFβ) signaling, which is important for the synthesis of type I procollagen [65,66]. TGFβ is a multifunctional cytokine that controls a multitude of processes, including tissue homeostasis and repair, differentiation of immune cells, cell growth, and apoptosis. The diversity of TGFβ responses is shaped by the two receptors TβR-I and TβR-II, harboring serine/threonine kinase activity [67]. Briefly, TGFβ binding induces clustering of both receptors leading to an interaction with adapter proteins, such as Smad anchor for receptor activation, and a recruitment of downstream mediators, in particular, Smad2, which is subsequently phosphorylated by TβR-I. Activated Smad2 recruits Smad4 to form a complex that translocates to the nucleus, binds to specific DNA motifs in the enhancer region of target genes and induces their transcription. A negative regulator of TGFβ signaling is Smad7, which disturbs Smad2/Smad4 complex formation by interacting with TβR-I and inhibiting Smad2 phosphorylation [67]. Both, exposure to UV radiation as well as to tobacco smoke extract interferes with TGFβ signaling and thereby reduces collagen synthesis. Whereas UV irradiation reduces TβR-II expression and increases Smad7 production [68,69,70], tobacco smoke extract blocks TGFβ signaling in skin fibroblasts by inducing a latent non-functional form of TGFβ_1_ and down-regulating TβR levels [71]. As outlined below, members of the transcription factor family AP-1 seem to play a crucial role for both, induction of ECM-degrading MMPs and interference with TGFβ signaling [61,69].

In the skin, as well as in several extra-cutaneous cells and tissues [72,73], MMP-1 expression was found being inducible by several AHR ligands. Specifically, TCDD treatment of normal human epidermal keratinocytes [74], exposure of human keratinocytes and fibroblasts with PAH-containing tobacco smoke extract [64], FICZ treatment of human normal dermal fibroblasts [75], and UVB irradiation of human skin in vivo [33], increased MMP-1 expression and activity in an AHR-dependent manner. The TCDD-stimulated induction of MMP-1 was further increased by a co-treatment of the keratinocytes with all-*trans* retinoic acid, which was mediated through two AP-1 binding sites in the MMP-1 promoter [74]. Functional AP-1 binding sites responsible for MMP-1 induction in response to various stress factors, including reactive oxygen species (ROS), UV radiation, and phorbol ester, have been previously identified in the MMP-1 gene promoter [76,77,78]. Also, treatment of human keratinocytes with the pro-inflammatory cytokine IL-1β transactivated EGFR-MEK-ERK signal transduction and downstream AP-1 activity to induce MMP-1 expression [79]. Interestingly, TCDD is known to induce IL-1β expression in human keratinocytes [80] and several environmental AHR agonists, such as TCDD and PAHs, may induce ROS formation by stimulating CYP1, aldo-keto reductase, or NADPH oxidase activities [81,82,83,84]. In addition, in co-exposure scenarios, PAHs and FICZ may serve as a photosensitizer for UVA radiation, resulting in a profound generation of ROS and associated oxidative damage and signaling responses [85,86,87]. In FICZ-treated fibroblasts, the usage of pharmacological inhibitors confirmed that the AHR-dependent upregulation of MMP-1 and MMP-3 is mediated through MEK-ERK signaling [75], pointing to an involvement of AP-1. Hence, even though the underlying mechanistic details remain to be elucidated, cutaneous AHR signaling is functionally involved in stimulating the production and release of MMP-1, potentially resulting in collagen breakdown and wrinkle formation. To make the story even more complex, several studies have shown that AHR activation affects different components of the plasminogen activation system [88], which cleaves pro-MMPs into their active form [89]. Briefly, the serine protease urokinase plasminogen activator (uPA) binds to its cell-surface receptor, resulting in the cleavage of plasminogen to plasmin. Plasmin cleaves and thereby activates several pro-MMPs as well as other ECM proteins. This protease system is controlled by two proteins, plasminogen activator inhibitor (PAI)-1 and PAI-2 (also known as Serpin E1 and Serpin B2), which block the uPA-mediated cleavage of plasminogen. In various keratinocyte cell-lines, TCDD was found to induce PAI-2 transcription, whereas it may enhance uPA levels through a post-transcriptional mechanism [80,90,91]. Moreover, by using transient RNAi, we have demonstrated that UVB exposure of human NCTC 2544 keratinocytes leads to an AHR-dependent transcriptional induction of PAI-2 [92]. Whether AHR-driven alterations of the plasminogen activation system are of functional relevance for extrinsic skin aging has not been investigated so far.

As already indicated above, AHR signaling may not only affect ECM degradation but also TGFβ-mediated procollagen synthesis. TGFβ stimulates dermal fibroblasts to transform to α-smooth muscle actin-expressing myofibroblasts, which produce type I procollagen [93,94]. Studies on fibroblasts from AHR-null mice revealed that these cells proliferate slower, express higher levels of TGFβ_1_ and ECM-related genes and secrete more TGFβ_1_ into the culture medium. Interestingly, overexpression of Smad7 reversed these effects and, as compared to AHR-proficient cells, restored proliferation rate and gene expression profile [95]. The functional relevance of the link between AHR status and TGFβ level was illustrated in a mouse wound healing model [96]. Wounds in AHR-null mice exhibited an increase in fibroblast numbers and elevated collagen content. Accordingly, AHR-null fibroblasts secreted higher levels of active TGFβ that stimulated keratinocyte migration, probably by sequentially over-activating the TGFβ signaling pathway and stimulating procollagen synthesis, finally leading to a faster wound healing in the AHR-null neo-epithelium [96]. Masutaka Furue and coworkers also reported that exposure of dermal fibroblasts to FICZ and kynurenine, another endogenous but less potent AHR agonist [97], interfered with TGFβ-regulated collagen homeostasis [98]. However, RNAi-mediated silencing of AHR did not affect the interference with collagen metabolism, suggesting that these effects occurred in an AHR-independent manner [98]. Thus, even though numerous studies confirmed the existence of a multifaceted, cell- and tissue-specific, and evolutionary conserved crosstalk between AHR and TGFβ signaling [99,100], the role of AHR in TGFβ-related collagen synthesis and associated consequences for ECM composition remain to be elucidated. For instance, assessing AHR’s role in the AP-1-dependent upregulation of Smad7, the endogenous inhibitor of TGFβ signaling, in UV-irradiated skin [69], would shed further light on this issue.

Not only PAH-loaded particulate matter but also gaseous constituents of ambient air pollution have been linked to skin aging [101]. A recent study, for instance, provides first epidemiological evidence that ozone exposure contributes to coarse wrinkle formation independently from further environmental risk factors [102]. Most likely, these effects are triggered by ozone-derived oxidative stress and the associated induction of MMPs and pro-inflammatory responses, for instance, increased NF-κB activity and COX-2 expression [103,104,105]. Interestingly, exposure of human primary keratinocytes to ozone resulted in nuclear translocation of AHR and elevated expression levels of CYP1 isoforms [106]. Moreover, ozone treatment stimulated EGFR phosphorylation and activation of downstream phosphoinositide 3-kinase and protein kinase B/AKT as well as MAPKs in an AHR-dependent manner. Thus, AHR signaling, at least in part, might mediate the detrimental effects of ozone by a yet unknown molecular mechanism.

### 2.2. Skin Pigmentation and Lentigines

Besides chloracne, accidental and occupational exposure to dibenzo-*p*-dioxins, dibenzofurans, dioxin-like polychlorinated biphenyls, and related halogenated aromatic hydrocarbons (e.g., in Yusho, Japan, 1968; Seveso, Italy, 1976; and Yucheng, Taiwan, 1979) causes a brownish hyperpigmentation of the skin [107,108,109], implying that an over-activation of the AHR system may affect melanocyte proliferation and/or function. Indeed, exposure of human melanocytes to TCDD resulted in an AHR-dependent activation of the melanogenic pathway, i.e., induction of tyrosinase activity and a subsequent increase in melanin content [27]. These effects were not associated with enhanced melanocyte proliferation but most likely due to an increased expression of pigmentation-relevant genes encoding tyrosinase and tyrosinase-like protein 2. In the context of extrinsic skin aging, it has been proposed that aberrant pigmentation of the skin of smokers results from AHR activation [71]. Accordingly, the treatment of melanocytes with tobacco smoke extracts led to an increased expression of microphthalmia-associated transcription factor (MITF), indicating melanocyte activation, which was blocked upon transient AHR-targeted RNAi [110]. In agreement with these in vitro findings, UVB irradiation of AHR-null mice resulted in a significantly weaker tanning response as compared to their wild type littermates [111]. Differences in tanning responses of AHR-null versus AHR-proficient mice were, however, not due to differences in tyrosine activity but to alterations in melanocyte numbers: Wild type mice exhibited a stronger increase in the number of dihydroxyphenylalanin-positive melanocytes upon irradiation than their AHR-deficient littermates. The lower number of melanocytes in AHR-null mice was associated with a significantly reduced expression of stem cell factor-1 and c-kit, which are both critical in regulating melanocyte differentiation and proliferation [111]. The other way round, dysregulation of epidermal AHR signaling, is associated with vitiligo, a depigmentation disorder of the skin associated with progressive loss of melanocytes [31,112]. Interestingly, most of the therapeutic measures taken to re-pigment vitiligo lesions, i.e., irradiation with narrow-band UVB or photochemotherapy (treatment with 8-methoxypsoralen or khellin and subsequent UVA exposure), have at least the potential to activate AHR signaling [16,113,114].

Finally, several epidemiological studies link the occurrence of lentigines on foreheads and cheeks of Caucasian [58] and Chinese [101] individuals to air pollution exposure. The first study, which established a connection between air pollution and skin ageing, was the SALIA study (SALIA, a study on the influence of air pollution on lung function, inflammation, and aging). Results from this study on 402 Caucasian women (aged 70–80 years), indicated that soot and traffic-related particulate matter contribute to an enhanced occurrence of pigment spots and, to a lesser extent, with skin wrinkling in the face [58]. These findings were validated in subsequent studies in Chinese populations, which were performed in two areas in Beijing with high and low levels of airborne particular matter [115]. High levels of particular matter were significantly associated with skin aging and senile lentigines formation [115]. As soot and traffic-related particular matter are rich in PAHs, it has been proposed that particle-bound PAHs and their cellular sensor molecule, namely AHR, are causally involved in air pollution-induced skin pigmentation as well [2]. In fact, extracts form airborne particulate matter and diesel exhaust particles have been proven to activate AHR and downstream signaling in various cutaneous and extra-cutaneous test systems [116,117,118].

## 3. AHR and Skin Cancer

Due to the accumulation of genomic alterations induced by environmental genotoxicants, in particular, UV radiation, the risk for developing skin cancer increases with age. NMSC, including basal cell carcinoma (BCC) and squamous cell carcinoma (SCC), is the most frequent malignancy in humans [7,9]. Current figures from the US are alarming, estimating the total number of NMSC in the US population at ~5.4 million affecting ~3.3 million individuals [7]. In contrast, malignant melanoma represents less than 5% of all diagnosed skin cancers, but, due to aggressive metastasis, it accounts for the vast majority of skin cancer deaths. The American Cancer Society predicts that in 2019, 96,480 individuals in the US will be diagnosed with malignant melanoma and 7,230 people will die from this devastating disease [119]. Importantly, researchers and authorities predict a further increase of NMSC as well as melanoma incidence within the next years. This is mainly due to the continuously growing number of elderly individuals in the general population as well as to the still unbroken popularity of tanned skin among younger generations [9,120]. Because skin cancer is not only a growing medical problem but also a substantial economic burden to health care systems [120,121], there is an urgent need for novel preventive and therapeutic measures.

To the best of our knowledge, neither a role for AHR in the pathogenesis of BCC nor an interaction of AHR with the sonic hedgehog signaling pathway, whose mutational impairment plays a key role in the pathogenesis of BCC [5], has been reported so far. In the following, we, therefore, focus on environmentally induced AHR signaling and its relevance for the development and progression of SCC and malignant melanoma.

### 3.1. Squamous Cell Carcinoma

The development of cutaneous SCCs is a multistep process, involving initiating and promoting events [122]. These include DNA damage and failure of appropriate cell rescue (DNA repair) or cell death (apoptosis) responses, the suppression of anti-tumor immune responses, and the clonal expansion of malignant cells [122,123]. Besides UVB radiation as the major risk factor for the development of SCCs [7,122], exposure to environmental, occupational, and lifestyle-related chemicals, especially PAHs, may contribute to SCC genesis [124].

To provoke effects at a cellular level, UVB radiation needs to be absorbed by chromophores to convert its physical into chemical energy. The most important chromophore for UVB radiation is the DNA [125]. In addition, other cellular components, in particular, aromatic amino acids such as tryptophan [29], can absorb UVB photons and contribute to the generation of the UVB stress response in the epidermal compartment [126,127]. The DNA damage-dependent part of this response is initiated by the UVB-induced formation of two photoproducts between adjacent pyrimidine bases: cyclobutane pyrimidine dimers (CPD) and pyrimidine (6-4) pyrimidone photoproducts [125]. Although both DNA photoproducts are highly mutagenic, CPDs are considered as being mainly responsible for skin photocarcinogenesis [128]. In mammals, UVB-induced DNA photoproducts are removed by nucleotide excision repair (NER) [129]. In case DNA damage is too severe or NER fails, damaged keratinocytes will initiate apoptosis in order to maintain tissue integrity and avoid mutagenesis [130].

There is ample evidence that AHR-dependent processes contribute to UVB radiation-induced photocarcinogenesis. In epidermal keratinocytes, absorption of UVB rays by tryptophan leads to the formation of FICZ and related photoproducts, which bind to AHR and activate downstream signaling pathways [16,29]. Genes that are upregulated in an AHR-dependent manner in UVB-exposed keratinocytes encode for xenobiotic-metabolizing enzymes, in particular CYP1A1 and CYP1B1 [16,131], as well as for pro-inflammatory COX-2 and C-X-C motif chemokine 5 (CXCL5) and related chemotactic factors [16,132]. The induction of CYP1 isoforms results in a rapid metabolism of AHR-activating FICZ [133], which probably attenuates AHR signaling when UVB irradiation ends. However, in situations of intense exposure, UVB radiation-triggered CYP1 activation may cause ROS formation and associated oxidative damage of macromolecules [83,84]. COX-2 is well-known to play a critical role in UVB-induced SCC development by generating pro-inflammatory and anti-apoptotic arachidonic acid metabolites that promote tumor growth [134]. In addition, the discussed AHR-dependent upregulation of MMPs in UVB-exposed skin may contribute to cancer progression by fostering processes such as tumor cell migration and invasion [61]. Gene expression profiling of healthy human skin, actinic keratosis, and invasive SCCs indeed identified AHR signaling as the second most significantly regulated signature gene set for invasion [135]. Hence, it was postulated that cutaneous AHR signaling pathways contribute to the UVB-induced development of keratinocyte-derived skin cancers [126]. Studies from our laboratory strongly support this notion by showing that in UVB-irradiated epidermal keratinocytes, AHR inhibits both the NER-mediated removal of CPDs and the initiation of programmed cell death [92,136]. Notably, enforced proteolysis of the cyclin-dependent kinase inhibitor and tumor suppressor protein p27^KIP1^ in response to AHR activation is critically involved in the repression of both cellular defense mechanisms [136] and associated with keratinocyte proliferation [92,137]. Accordingly, AHR inhibition stabilized cutaneous p27^KIP1^ protein levels, increased CPD repair, elevated apoptosis of remaining damaged keratinocytes, and reduced UVB radiation-induced SCC formation in SKH-1 hairless mice by approximately 50%, thus providing evidence that AHR critically contributes to photocarcinogenesis [136]. The dramatic reduction in UVB radiation-induced SCC formation in AHR-null mice may also be due to enhanced anti-tumor immune responses in these animals. In fact, CPDs are the major trigger for UVB radiation-induced immunosuppression [138], and an accelerated repair of these lesions may thus dampen the extent of immunosuppression [139,140]. In addition, AHR activation by either UVB irradiation or chemical AHR ligands switched antigen-presenting dendritic cells from a stimulatory into a regulatory phenotype thereby leading to the induction of regulatory T cells independently from DNA damage [141,142]. Notably, the AHR-dependent production of IL-2, whose gene promotor harbors functional xenobiotic-responsive elements [143], was identified as being critical for the immunosuppressive properties of AHR [141]. In addition, AHR activation stimulated the expression of indolamine-2,3-dioxygenases (IDO) [141], a tryptophan-metabolizing and kynurenine-generating enzyme, which is known to promote immunosuppressive effects by activating regulatory T cells and suppressing the functions of effector T cells and natural killer cells [144].

In conclusion, the UVB radiation-induced generation of SCCs seems to rely, at least to a major extent, on AHR activation and downstream modulation of DNA damage-dependent responses. The translational relevance of these findings is highlighted by a recent two-stage genome-wide association study, which identified AHR as a novel susceptibility locus for SCC in humans [145].

Particularly in occupational settings, exposure to PAHs, for instance, present in soot, tar, and bitumen, is considered as being a major risk factor for cutaneous SCC development in humans [146]. Also, the elevated risk of current smokers to develop cutaneous SCCs (but not BCCs) [147,148] is largely attributed to PAHs along with nitrosamines and aromatic amines present in tobacco smoke [149]. The carcinogenic potential of PAHs is closely linked to AHR signaling as it is unleashed upon metabolic activation by the AHR-dependent phase-I monooxygenases CYP1A1, CYP1A2, and CYP1B1, which are present and inducible in human skin [150]. These enzymes oxidize PAHs to enhance their water solubility and enable conjugation to hydrophilic moieties, such as activated sugar, sulfate, or glutathione, by phase-II drug-metabolizing enzymes. If the capacity of these conjugating enzymes is exhausted, reactive phase-I metabolites may attack the DNA and cause mutations. Interestingly, AHR inhibition protects against the carcinogenicity of PAHs that are primary substrates for CYP1A1, but not against the genotoxic effects provoked by PAHs that are predominantly metabolized by CYP1B1. For instance, benzo(*a*)pyrene (BaP) is sequentially metabolized by CYP1A1 and microsomal epoxide hydrolase 1 to BaP-7,8-dihydrodiol-9,10-epoxide, an highly carcinogenic compound [151,152]. Since CYP1A1 expression is abolished in AHR-null mice, these animals are resistant toward BaP-induced skin carcinogenesis [153]. Along the same line, gene targeting of ARNT in the epidermis of adult mice completely prevented the development of BaP-initiated skin tumors [154]. In contrast, 7,12-dimethylbenz(*a*)anthracene (DMBA) is metabolized by CYP1A1, CYP1B1, and microsomal epoxide hydrolase 1 [155,156]. Importantly, the carcinogenic potential of DMBA strongly depends on which CYP1 isoform is predominantly expressed in the exposed cell population or tissue. In contrast to CYP1A1-mediated DMBA metabolism, which favors detoxification, CYP1B1-mediated metabolism of DMBA results in an enhanced formation of DMBA-trans-3,4-diol, which is subsequently transformed to the high mutagenic DMBA-3,4-diol-1,2-epoxide [155,156]. A study from the Girardi laboratory revealed that in keratinocytes CYP1A1 is expressed significantly higher than CYP1B1, whereas the latter is the predominating CYP1 family member in Langerhans cells (LCs) [157]. Experimental depletion of epidermal LCs protected the respective mice against DMBA-induced skin carcinogenesis, indicating that the generation of DMBA-3,4-diol-1,2-epoxide is mainly mediated by LCs [157]. Expression of CYP1B1 is not only controlled by AHR, but also by other transcription factors, such as estrogen receptor-α [158]. Accordingly, the CYP1B1 expression level in AHR-null mice is still high enough to toxify DMBA [159]. Hence, DMBA-treated AHR-null mice develop SCC in comparable amounts to AHR wild-type mice [159]. Finally, AHR-null mice were shown to be largely protected against SCC development induced by extracts of exposure-relevant airborne particulate matter [118].

Apart from UVA-related phototoxicity of PAHs [86,87], the AHR-dependent upregulation of CYP1 isoforms in UVB radiation-exposed skin may sensitize cells to PAH-mediated DNA adduct formation, thereby enhancing the risk of respectively exposed people, e.g., roofers and roadmen, to develop SCCs. In fact, a study assessing the influence of UVB irradiation and crude coal tar treatment on cutaneous CYP1 enzyme activity and BaP metabolism revealed that crude coal tar, as well as UVB exposure alone, induced CYP1 activity and BaP oxidation. Interestingly, sequential treatment studies, i.e., first UVB exposure followed by coal tar application and vice versa, turned out that only the sequence coal tar > UVB (and not UVB > coal tar) resulted in an additive induction of CYP1 activity, BaP metabolism, and BaP-DNA binding [160]. Results from an in vitro study showing that UVB irradiation or FICZ treatment of human HaCaT keratinocytes led to an increased expression of CYP1A1 and CYP1B1, which predisposed cells to an enhanced DNA adduct formation in response to BaP exposure [161], do not confirm this sequence-dependency. This might be due to the transient nature of the UVB-triggered AHR activation and CYP1 induction and respective study-related differences in treatment time. However, recent studies assessing the metabolic activation of PAHs and PAH mixtures in human skin explants revealed that a co-exposure to the full solar UV spectrum strongly inhibited PAH metabolism [162,163]. This to some extent surprising observation might be due to either a UVA-related generation of ROS and associated inhibition of nuclear factor-1, a general transcription factor that interacts with AHR at the proximal promoter of CYP1A1 [164], or to a UV-triggered production of cytokines, such as tumor necrosis factor-α or IL-1β, that may repress CYP1A1 transcription over time [19,165]. Further studies elucidating the putative co-carcinogenic potential of UV radiation and PAHs are necessary to enable proper risk assessments.

### 3.2. Malignant Melanoma

Results from epidemiological studies indicate that residential and occupational exposure of humans to pesticides, which often contain AHR-modulating chemicals, increases the risk for cutaneous malignant melanoma [166,167,168]. A positive association between dioxin exposure and incidence of malignant melanoma (and prostate cancer), for instance, was reported from Operation Ranch Hand veterans, who sprayed TCDD-contaminated herbicides, especially Agent Orange, during the Vietnam War [168]. Notably, chemical carcinogenesis studies on mice have demonstrated that TCDD is a potent tumor promoter in tissues, such as liver and skin [169,170], suggesting that it mainly contributes to melanoma development by fostering the survival and proliferation of initiated melanocytes. In support of this notion, genome-wide association studies more recently identified AHR as a susceptibility locus, which is involved in both the skin pigmentation/decreased tanning response and increased risk for melanoma [171,172,173].

Gene expression analyses revealed that AHR is highly expressed in some melanoma cell lines [174]. In different experimental melanoma models, making use of cell culture approaches and in vivo models, it was shown that AHR activation can modulate several central hallmarks of cancer. However, contradictory effects have been observed. For example, TCDD exposure of human A2058 melanoma cells induced the expression of MMP-1, MMP-2, and MMP-9, which was associated with enhanced invasive growth of melanoma cells in vitro [175]. In contrast, AHR expression was not found in highly migratory and invasive C8161 cells, and ectopic overexpression of AHR in C8161 cells reduced their migratory capacity [176]. Silencing of AHR via the introduction of targeting small hairpin RNAs resulted in growth deficiency in AHR-high human melanoma cell lines, IPC-298, and SK-MEL-2 [177]. Experiments with B16 melanoma cells and tumor transplantation studies in mice indicated that AHR has anti-tumorigenic properties in tumor cells but pro-tumorigenic functions in the tumor stroma [176]. AHR signaling contributes to the development of a tumor-promoting microenvironment in the surrounding stroma by stimulating signaling pathways and mediators that facilitate angiogenesis and cancer cell motility (including vascular endothelial growth factor and TGFβ) [178,179].

In the last few years, a role for AHR has emerged in regulating response and resistance to novel targeted therapies for metastatic melanoma. In NRAS mutated cell lines, the response to the MEK inhibitor PD0325901 correlated with the expression of AHR [177]. Mechanistically, this might be explained by a direct interaction of the MEK inhibitor with AHR activity. In fact, several structurally related protein kinase inhibitors, including other MEK inhibitors, such as PD98059 and U0126, have been previously identified to serve as AHR ligands and modulate downstream responses [180]. Moreover, a direct interaction of AHR with the BRAF inhibitor vemurafenib has been described in melanoma cells [181] as well as in T cells and keratinocytes [182]. Activation of AHR conveyed melanoma cells with resistance to BRAF inhibitors. Accordingly, inhibition of AHR sensitized melanoma cells to targeted therapy [181].

AHR also appears to be involved in modulating responses to novel immunotherapies with antibodies that block immunoregulatory molecules, such as PD-1 and PD-L1. In brain tumors, it was first shown that the tryptophan metabolite and AHR ligand kynurenine are produced via the metabolic enzymes IDO or tryptophan-2,3-dioxygenases (TDO) and can inhibit immune cell proliferation and functions [183]. Similar results were reported in other tumor entities, such as lung and breast cancer [184,185].

The production of IFN-γ by tumor-specific T cells is a key effector mechanism of immunotherapies. Interestingly, IFN-γ treatment-induced dormancy in B16 melanoma cells in an IDO- and AHR-dependent manner [186]. Inhibition of both IDO and AHR induced apoptosis of dormant melanoma cells by activating signal transducer and activator of transcription-3 and stimulating p53 expression [186,187]. Additionally, AHR stimulated the upregulation of PD-1 and, accordingly, AHR inhibition increased the efficacy of antitumor adoptive T cell therapy [188]. AHR signaling was recently shown to enhance PD-L1 expression on tumor cells and decreased response to immune checkpoint blockade in lung cancer [189]. It is hypothesized that the IDO/TDO-kynurenine-AHR axis is involved in developing therapy resistance to immunotherapies and might represent a promising additional target [190].

## 4. Conclusions

**Figure 2:**
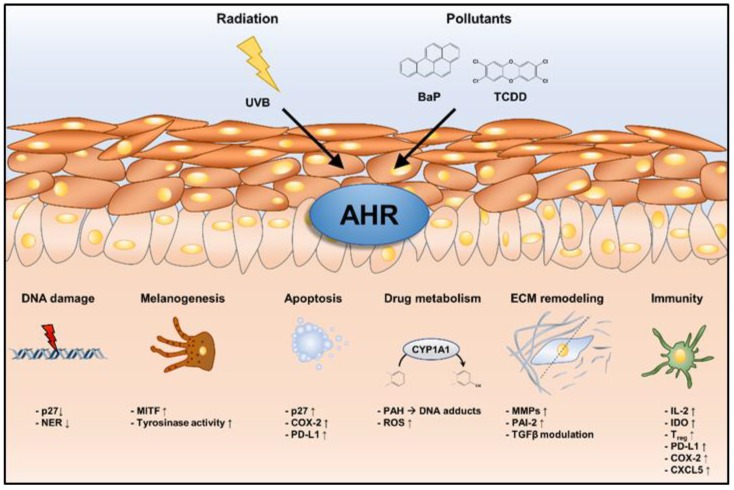
Cutaneous cellular effects in response to environmentally induced sustained AHR activation. Sustained activation of AHR by chronic exposure of the skin to either UVB radiation or chemical pollutants may affect a multitude of cellular processes that contribute to extrinsic skin aging and the development and progression of skin cancer.

Various environmental stressors, including UV radiation and PAH-rich airborne particulate matter and tobacco smoke, are capable of shaping AHR activity and downstream signaling responses in the skin, thus contributing to the development and/or progression of several aging traits and various types of skin cancer (Figure 2). Due to the demographic change in Western societies and the associated increasing prevalence of these chronic disorders and diseases of the skin, in particular, skin cancer, present a major medical but also economic burden for our health care systems. We believe that safe, transient acting and good penetrating AHR inhibitors are promising tools to induce protective and possibly therapeutic responses in the skin. Whereas the chemopreventive potential of AHR antagonists against PAH-induced skin cancers is mainly due to a decrease in CYP1A enzyme activity and associated metabolic activation, these compounds may alter ECM remodeling, DNA repair and apoptosis to prevent or delay skin wrinkling and photocarcinogenesis.

AHR-driven melanocyte-related signs of skin aging, in particular, lentigines, are probably due to a direct influence on melanin synthesis, which is also supported by the fact that AHR signaling is disturbed in vitiligo and most of the current strategies to induce re-pigmentation at least potentially activate AHR. For malignant melanoma, AHR’s role is less clear, which emphasizes the urgent need for additional mechanistic and clinical-oriented studies, for instance, assessing AHR’s impact on DNA repair, apoptosis, and respective consequences for melanoma initiation. Recent studies indicate that AHR inhibition might be a suitable strategy to enhance the efficacy of immunotherapeutic applications against malignant melanoma and, in addition, AHR modulation may affect melanoma resistance against protein kinase inhibitors frequently used in the clinical routine. Hence, we conclude that the development of transient AHR antagonists that are suitable for topical application in humans and their in-depth characterization in preclinical models are important in terms of prevention and treatment of environmentally induced skin aging and skin carcinogenesis. One candidate compound may be E/Z-2-benzylindene-5,6-dimethoxy-3,3-dimethylindan-1-one, a registered cosmetic ingredient that was shown to efficiently attenuate AHR-induced signaling responses in UVB-irradiated human skin [33].

## Abbreviations

AHR	aryl hydrocarbon receptor
ARNT	aryl hydrocarbon receptor nuclear translocator
BaP	benzo(a)pyrene
BCC	basal cell carcinoma
CPD	cyclobutane pyrimidine dimer
COX-2	cyclooxygenase-2
CXCL5	C-X-C motif chemokine 5
CYP	cytochrome P450
DMBA	7,12-dimethylbenz(a)anthracene
ECM	extracellular matrix
EGFR	epidermal growth factor receptor
FICZ	6-formylindolo[3,2-b]carbazole
IDO	indolamine-2,3-dioxygenase
IFN	interferon
LC	Langerhans cell
MAPK	mitogen-activated protein kinase
MITF	microphthalmia-associated transcription factor
MMP	matrix metalloprotease
NER	nucleotide excision repair
NF-κB	nuclear factor-kappa B
PAH	polycyclic aromatic hydrocarbon
PAI	plasminogen activator inhibitor
ROS	reactive oxygen species
SCC	and squamous cell carcinoma
TCDD	2,3,7,8-tetrachlorodibenzo-*p*-dioxin
TDO	tryptophan-2,3-dioxygenases
TGFβ	transforming growth factor-β
uPA	urokinase plasminogen activator
UV	ultraviolet
